# Development of Genetic Markers Linked to Straighthead Resistance through Fine Mapping in Rice (*Oryza sativa* L.)

**DOI:** 10.1371/journal.pone.0052540

**Published:** 2012-12-28

**Authors:** Xuhao Pan, Qijun Zhang, Wengui Yan, Melissa Jia, Aaron Jackson, Xiaobai Li, Limeng Jia, Bihu Huang, Peizhou Xu, Fernando Correa-Victoria, Shigui Li

**Affiliations:** 1 Rice Research Institute, Sichuan Agricultural University, Chengdu, China; 2 Rice Research and Extension Center, University of Arkansas, Stuttgart, Arkansas, United States of America; 3 USDA-ARS, Dale Bumpers National Rice Research Center, Stuttgart, Arkansas, United States of America; 4 Institute of Food Crops, Jiangsu Academy of Agricultural Sciences, Nanjing, China; 5 State Key Lab of Rice Biology, Institute of Nuclear-Agriculture Sciences, Zhejiang University, Hangzhou, China; 6 The Institute of Virology and Biotechnology, Zhejiang Academy of Agriculture Sciences, Zhejiang, China; 7 University of Arkansas at Pine Bluff, Pine Bluff, Arkansas, United States of America; 8 RiceTec, Inc., Alvin, Texas, United States of America; New Mexico State University, United States of America

## Abstract

Straighthead, a physiological disorder characterized by sterile florets and distorted spikelets, causes significant yield losses in rice, and occurs in many countries. The current control method of draining paddies early in the season stresses plants, is costly, and wastes water. Development of resistant cultivar is regarded as the most efficient way for its control. We mapped a QTL for straighthead resistance using two recombinant inbred line (RIL) F_9_ populations that were phenotyped over two years using monosodium methanearsonate (MSMA) to induce the symptoms. One population of 170 RILs was genotyped with 136 SSRs and the other population of 91 RILs was genotyped with 159 SSRs. A major QTL *qSH-8* was identified in an overlapping region in both populations, and explained 46% of total variation in one and 67% in another population for straighthead resistance. *qSH-8* was fine mapped from 1.0 Mbp to 340 kb using 7 SSR markers and further mapped to 290 kb in a population between RM22573 and InDel 27 using 4 InDel markers. SSR AP3858-1 and InDel 11 were within the fine mapped region, and co-segregated with straighthead resistance in both RIL populations, as well as in a collection of diverse global accessions. These results demonstrate that AP3858-1 and InDel 11 can be used for marker-assisted selection (MAS) for straighthead resistant cultivars, which is especially important because there is no effective way to directly evaluate straighthead resistance.

## Introduction

Straighthead is a physiological disorder in rice that is characterized by spikelet sterility resulting in blank panicles which remain upright at maturity. Straighthead can result in almost complete loss of grain yield [1], [2], and is occurring with increasing frequency in Arkansas where about 50% of USA rice is produced [3]–[5]. For example, yield loss up to 94% has been observed in Cocodrie [1], a major commercial cultivar in the southern USA [6]. Straighthead is also a global threat to rice production and has been reported in Portugal [7], Thailand [8], Japan [9], Australia [10], Argentina [11], and Brazil (Correa-Victoria, pers. comm.).

A water management practice that is called ‘Draining and Drying’ (D&D) is the only recommended method to prevent straighthead in rice in the USA [3], [12]. Rice fields are drained about 2 weeks after a permanent flood, dried until rice leaves exhibit drought stress symptoms, and then re-flooded [2], [13], [14]. Currently, the D&D method is applied to more than one third of the rice acreage in Arkansas as a preventative measure and wastes some 150 million m^3^ of irrigation water each year [15]. Once straighthead occurs in a field, growers will keep using the D&D method because of unaffordable consequences. This method of straighthead prevention is costly and wasteful of natural resources, and results in drought-related yield loss.

Varietal resistance is regarded as the most efficient, economical, and environmentally friendly strategy for straighthead prevention [1], [2], [10]. The earliest attempt at breeding for straighthead resistance in the USA started in 1950s [16], but little progress had been made because the inheritance of straighthead resistance was not well understood and the identification of resistant germplasm was limited until 2002 [17]. Using a diversity panel of 547 germplasm accessions, Agrama and Yan [18] identified six simple sequence repeat (SSR) markers which significantly associated with straighthead resistance by association mapping.

Since the 1930’s, researchers in the USA have associated straighthead-like symptoms with the use of arsenic-based herbicides [19]. Thus, the breeding community has used soil incorporation of *As* in the form of MSMA as a common practice for evaluating rice susceptibility to straighthead [1], [10], [14], [20], [21]. Using this screening method, cultivars are rated on a 1 to 9 scale based upon the level of panicle distortion and sterility. Evaluation with MSMA is the only known method for consistent screening of straighthead susceptibility.

Linkage mapping is a classical approach in tagging genes responsible for valuable traits and has been widely used in crops [22]–[26]. Populations that are used for linkage mapping include recombinant inbred lines (RILs), F_2_ populations, backcross populations, near isogenic lines and diversity panels. In the present study, our objective was to identify and fine map QTL(s) associated with straighthead resistance using two RIL populations and to develop DNA markers to be used in the MAS for straighthead resistant cultivars.

## Materials and Methods

### Plant Materials

Two RIL F_9_ populations were constructed for linkage mapping, one from the cross between resistant cultivar Zhe733 (PI 629016) and susceptible cultivar R312 (PI 614959) and another from the cross between resistant cultivar Jing185 (PI 615205) and susceptible cultivar Cocodrie (PI 606331) [17]. Zhe733, R312 and Jing185 are introductions from China while Cocodrie is a widely grown USA cultivar, and all of them have been well characterized for straighthead responses previously [2]. Zhe733 and R312 belong to the *indica* sub-species while Cocodrie is *japonica*, and ‘Jing’ means *japonica* in Chinese. Our intention was to make crosses within subspecies i.e. *indica*/*indica* and *japonica*/*japonica* to avoid sterility due to subspecies incompatibility; however, a subsequent study indicated that Jing185 is an *indica* [12]. The Zhe733/R312 population was comprised of 170 F_9_ RILs while the Cocodrie/Jing185 cross ultimately produced only 91 F_9_ RILs as a result of the loss of some offspring due to incompatibility in the wide *japonica/indica* cross. After molecular markers for straighthead resistance were developed using the two RIL populations, a diversity panel was used to assess their effectiveness across a broad range of global germplasm. A set of 72 accessions were selected from the USDA rice core collection [18] based on their representation of global diversity and diverse straighthead responses.

### Phenotyping

Straighthead evaluation of the mapping populations was conducted under arsenic-amended soil conditions at Dale Bumpers National Rice Research Center near Stuttgart, Arkansas in 2008 and 2009. RILs of Zhe733/R312 and Cocodrie/Jing185 F_9_ populations were planted in single row field plots (0.62 m^2^) using a randomized complete block design with three replications. MSMA was applied to the soil surface at 6.7 kg ha^−1^ and incorporated prior to planting as previously described [2], [27]. The four parents of the two populations were repeatedly arranged in each field tier of 99 rows as controls. Field management was performed as described in [2], [27].

Straighthead was rated at maturity based on floret sterility and panicle emergence from the flag leaf sheath using a scale from 1 to 9 [2], where a score of 1 was normal plants with no apparent sterility (more than 80% grains developed) and 100% of the panicles emerged, and 9 represented short stunted plants with severe straighthead and no panicle emergence from the flag leaf sheath, thus complete absence of developed grains. Straighthead ratings were analyzed using ANOVA (analysis of variance) procedure of SAS software 9.1 version (SAS Institute Inc., Cary, NC). RILs with a straighthead rating of 4.0 or below were considered resistant based upon previous research which had demonstrated no yield loss at this level, while those with a rating of 6.0 or above were considered susceptible [2].

### DNA Extraction and Genotyping

DNA was extracted from each RIL and their parents following the CTAB method descried by Hulbert and Bennetzen [28]. We screened 521 and 473 genome-wide SSR markers obtained from Gramene (http://www.gramene.org) for polymorphism in the Zhe733/R312 and Cocodrie/Jing185 crosses, respectively. Polymorphic markers were used to construct linkage maps for each F_9_ RIL population.

For each marker, forward primers were labeled with fluorescent dyes (6FAM, NED and HEX) from Applied Biosystems (Foster City, CA) or Integrated DNA Technologies (Coralville, IA). MJ Research Tetrad thermalcyclers were used to amplify DNA (Waltham, MA) under the following PCR conditions: (1) initial denaturation at 94°C for 5 min; (2) 35 cycles at 94°C for 1 min, 55°C to 67°C (marker dependent) for 1 min and 72°C for 2 min; (3) final extension at 72°C for 5 min. The PCR products were pooled based on color and size range of the amplified products (typically three markers per run along with LIZ-labeled size standard), and the DNA was denatured by heating at 94°C for 5 min. An ABI 3730 DNA analyzer was used to separate samples according to the manufacturer’s instructions (Applied Biosystems, Foster City, CA). The size of each SSR fragment was estimated and the alleles were binned using GeneMapper® (Applied Biosystems, Foster City, CA).

### Linkage Mapping and Identification of Recombinant RILs

Straighthead ratings averaged over six observations (three replications in 2008 and 2009) for each RIL were used for the mapping. Linkage map was constructed using polymorphic markers by JoinMap 4.0 with the Kosambi mapping function. Qgene 4.3.8 was used to map QTL and estimate QTL parameters (effects and test statistics) with the composite interval mapping (CIM) method [29]. Qgene 4.3.8 with the permutation function was used to determine LOD scores. LOD ≥2.8 were claimed as QTL for straighthead resistance in Zhe733/R312 and 3.8 in Cocodrie/Jing185 with a probability level of 0.05. Once the straighthead-associated QTL were preliminarily identified, the RILs in which a recombination occurred within the target region for the putative QTL were selected for fine mapping.

### Fine Mapping

After the target region was located, all SSR markers within the region were searched using the Gramene database (http://www.gramene.org). The SSR markers were screened for parental polymorphism in each population and the polymorphic ones were used for fine mapping. In an effort to saturate the target region, additional InDel and SSR markers were developed using the MSU rice genome browser http://rice.plantbiology.msu.edu/cgi-bin/gbrowse/rice/. InDel markers were developed by comparing Nipponbare and 93–11 sequences in the targeted region. Primers were designed using the NCBI online primer design tool (http://www.ncbi.nlm.nih.gov/tools/primer-blast/index.cgi?LINK_LOC=BlastHome). New SSRs were named based on the BAC clone from which they were designed. The designed InDel and SSR markers were screened for parental polymorphism in each population and the polymorphic ones were used for further fine mapping.

### Identification of Markers Associated with Straighthead Resistance for Breeding

After fine mapping, the markers within the QTL region were verified for co-segregation of genotype with straighthead phenotype in both RIL populations. We excluded those RILs with an intermediate straighthead rating of 4.1–5.9. As a result, 160 and 79 RILs that were either straighthead resistance (rating≤4.0) or susceptibility (rating≥6.0) were selected for verification from Zhe733/R312 and Cocodrie/Jing185, respectively. Furthermore, these markers were applied to a global germplasm collection of 72 accessions for association with straighthead resistance. Those accessions originated from 28 countries with the most from China (22), followed by the Philippines and the USA. Forty were resistant to straighthead with rating 4 or less, and the remaining 32 were susceptible with straighthead ratings of 6 or more based on previous studies by Yan et al. [2] and [30]. Chi square (*χ^2^*) test for goodness of fit to independence was conducted for the co-segregation of genotype and phenotype. According to the Hardy-Weinberg equilibrium, H_0_ was that the marker genotype was independent from straighthead phenotype in each RIL or germplasm accession, and H_ A_ was that the marker genotype corresponded with straighthead phenotype in each RIL or germplasm accession.

## Results

### Straighthead Variation in the RIL Populations

**Figure 1:**
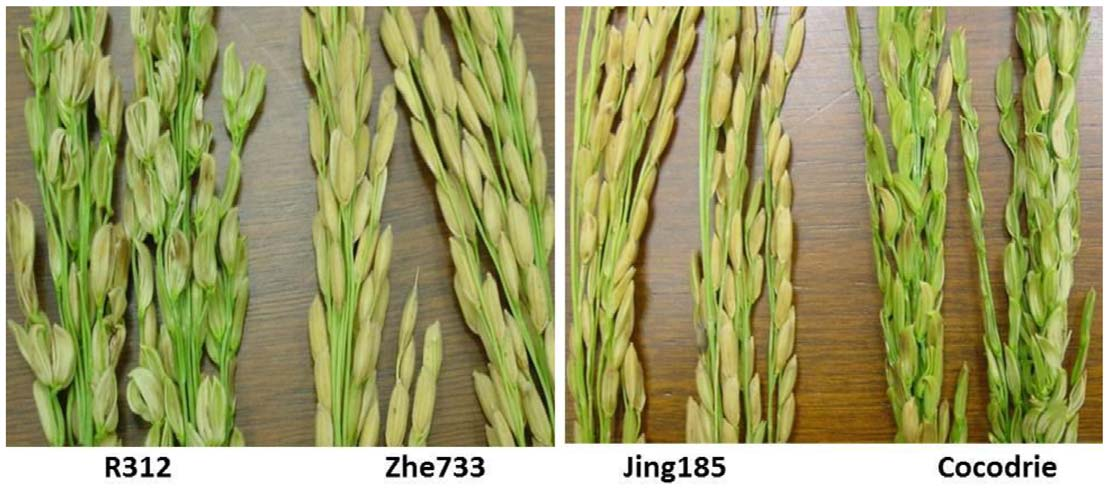
Straighthead phenotypes in parents of two mapping populations. Resistant parents Zhe733 and Jing185 had fully developed panicles while susceptible parents R312 and Cocodrie had panicles with severely distorted spikelets.

**Figure 2:**
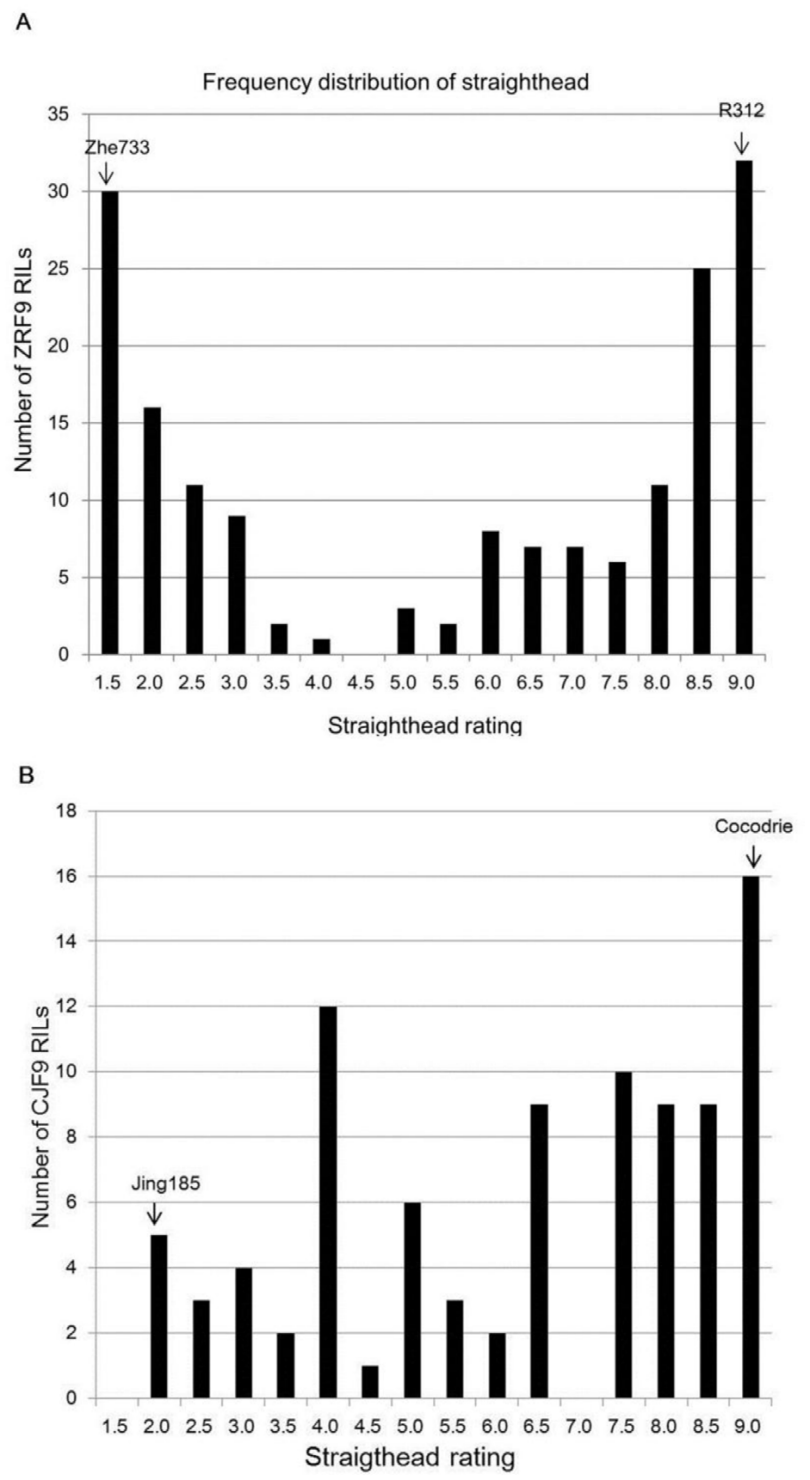
Distribution of RILs of two populations. Distribution of straighthead ratings among 170 Zhe733/R312 F_9_ RILs and two parents Zhe733 and R312 (A), and among 91 Cocodrie/Jing185 F_9_ RILs and two parents Cocodrie and Jing185 (B). Coefficient of variation was 16.7% and 13.1% in Zhe733/R312 and Cocodrie/Jing185 populations, respectively.

Resistant parents, Zhe733 and Jing185, were rated less than 2 with no straighthead symptoms, while susceptible parents, R312 and Cocodrie were rated more than 8 with severe straighthead symptoms (Fig. 1 and 2). Straighthead segregated across the full rating range from 1 to 9 in both F_9_ RIL populations, which validated the effectiveness phenotyping of straighthead using MSMA (Fig. 2). In Zhe733/R312 population, most RILs were rated as resistant (4.0 or below) or as susceptible (6.0 or above) (Fig. 2a). However, in the Cocodrie/Jing185 population (Fig. 2b), a smaller proportion of the RIL population (28.5%) was resistant than in the Zhe733/R312 population (40.6%). Over two years, straighthead had a coefficient of variation of 16.7% and 13.1% for Zhe733/R312 and Cocodrie/Jing185 populations, respectively. Although the variations due to RIL genotype, Year and RIL*Year were all significant, RIL took a great part of total variation for straighthead. In Zhe733/R312 population, 95.2%, 3.6% and 1.1% of the total variation were explained by RIL, RIL*Year and Year, respectively. Similarly in Cocodrie/Jing185 population, 91.0%, 7.8% and 1.1% of the total variation were explained by RIL, RIL*Year and Year, respectively.

### Construction of Linkage Map and QTL Analysis in Two Populations

**Figure 3:**
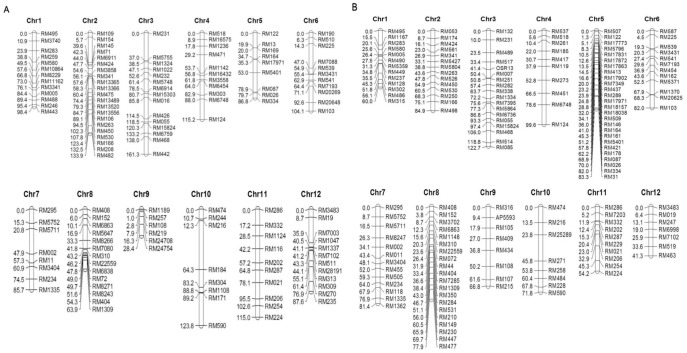
Linkage map constructed by polymorphic SSR markers in Zhe733/R312 population (A), and Cocodrie/Jing185 population (B). Number 1 to 12 represent chromosome number in rice genome.

For Zhe733/R312, 136 polymorphic SSR markers providing genome wide coverage approximately every 17.1cM were used for mapping (Fig. 3a). For Cocodrie/Jing185, 159 polymorphic SSRs providing genome wide coverage approximately every 5.8 cM were used (Fig. 3b).

**Figure 4:**
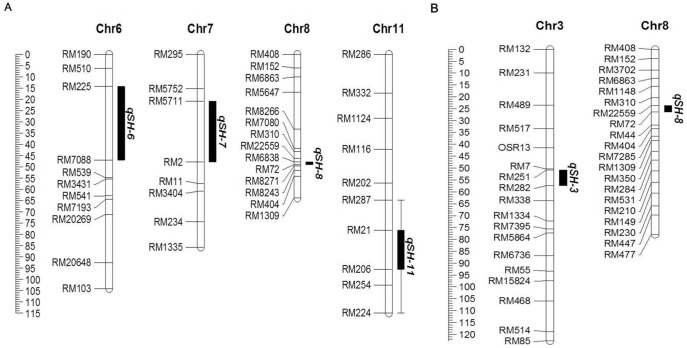
Straighthead-associated QTLs identified in Zhe733/R312 population (A), and Cocodrie/Jing185 population (B). Black bar shows the position of each QTL on rice chromosome (Chr).

Four QTL were identified to be associated with straighthead rating in Zhe733/R312 population on chromosomes (chr) 6, 7, 8 and 11 (Fig. 4a) (Table 1). The QTL on chr8 had the largest LOD (23.0), highest additive effect (−2.1) and smallest marker interval (1.0 cM) between RM6838 and RM72, and explained the most total variation (46%) for straighthead rating among the identified QTL. From Cocodrie/Jing185 population, two QTL were identified (Fig. 4b) (Table 1), one on chr3 (LOD = 3.8), and another on chr8 (LOD = 27.0). The chr8 QTL was within a 1.9 cM interval between RM22559 and RM 72, had a −2.1 additive effect, and explained 67% of total variation for straighthead rating. The chr8 QTL appeared to play a major role in straighthead resistance in both populations.

**Table 1:** QTLs identified in two recombined inbred line (RIL) F_9_ populations using Qgene 4.3.8.

Populations	Chr no.	QTLs	LOD^a^	Explained phenotype variation	Additive effect
Zhe733/R312	chr6	*qSH-6*	4.8	0.13	−1.9
	chr7	*qSH-7*	5.0	0.12	−1.7
	chr8	*qSH-8*	23.0	0.46	−2.1
	chr11	*qSH-11*	3.0	0.08	−1.1
Cocodrie/Jing185	chr3	*qSH-3*	3.8	0.18	0.7
	chr8	*qSH-8*	27.0	0.67	−2.1

a According to permutation function in Qgene, LOD≥2.8 was used to claim QTL associated with straighthead in Zhe733/R312 and LOD≥3.8 in Cocodrie/Jing185 using a probability level of 0.05.

**Figure 5:**
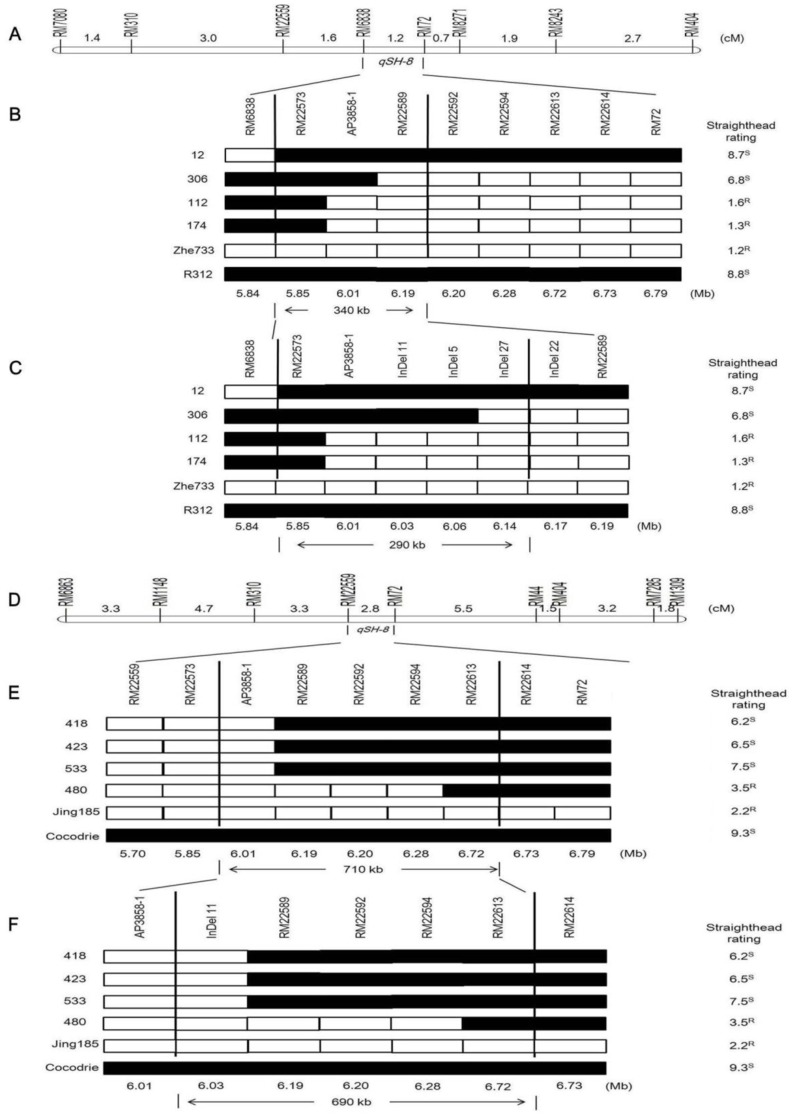
Fine mapping of *qSH-8* on chromosome 8 using Zhe733/R312 (A-C) and Cocodrie/Jing185 (D-F) F_9_ RIL populations. Genetic distance cM is the number between markers and physical distance is placed below markers, *qSH-8* in a 1.2 cM region between RM6838 and RM72 (A), a 340 kb region between RM22573 and RM22589 (B) and a 290 kb region between RM22573 and InDel 27 (C) in Zhe733/R312; and a 2.8 cM between RM22559 and RM72 (D), a 710 kb region between AP3858-1 and RM22613 (E), and a 690 kb region between InDel 11 and RM22613 (F) in Cocodrie/Jing185.

RM72 at 6.76 Mb was the most distal marker of the chr8 QTL identified in both populations. RM6838 in Zhe733/R312 and RM22559 in Cocodrie/Jing185 were physically located very close to each other at 5.85 Mb and 5.70 Mb, respectively. The overlapping intervals on chr8 identified in both populations indicate the presence of a major QTL at this location which we designated as *qSH-8* (Fig. 5a for Zhe733/R312 and 5d for Cocodrie/Jing185).

### Fine Mapping of *qSH-8*

Within the putative region of *qSH-8*, four recombinants (RIL12, 112, 174, and 306) were identified in Zhe733/R312 population (Table 2), and four (RIL418, 423, 480, and 533) were identified in Cocodrie/Jing185 as recombinants (Table 3). We obtained an additional 16 SSR markers in *qSH-8* region from the Gramene database and developed one additional SSR from the MSU rice genome browser, seven of which were polymorphic for parents of both populations (Table 4). According to the substitution mapping strategy described by Paterson et al. [31], mapping with recombinants using polymorphic SSRs narrowed down *qSH-8* region to an interval of 340 kb between RM22573 and RM22589 in Zhe733/R312 population (Fig. 5b), and 710 kb between AP3858-1 and RM22613 in Cocodrie/Jing185 population (Fig. 5e).

**Table 2:** Recombinants identified in the target region from population Zhe733/R312 between RM6838 and RM72.

	Genotype^a^			
RIL No.	RM6838	RM72	Straighthead rating^b^	Phenotype
12	a	b	8.67±0.52	Susceptible
112	b	a	1.58±0.89	Resistant
174	b	a	1.25±0.45	Resistant
306	b	a	6.83±1.33	Susceptible

a ‘a’ for resistant genotype of Zhe733, ‘b’ for susceptible genotype of R312.

b Straighthead rating using a 1–9 scale was averaged over 3 replications per year and 2 years for which the SD was estimated. Straighthead rating of 4 or below was resistant and 6 or above was susceptible.

**Table 3:** Recombinants identified in the target region from population Cocodrie/Jing185 between RM22559 and RM72.

	Genotype^a^			
RIL No.	RM22559	RM72	Straighthead rating^b^	Phenotype
418	a	b	6.17±0.75	Susceptible
423	a	b	6.50±0.84	Susceptible
480	a	b	3.50±1.38	Resistant
533	a	b	7.50±1.05	Susceptible

a ‘a’ for resistant genotype of Jing185, ‘b’ for susceptible genotype of Cocodrie.

b Straighthead rating using a 1–9 scale was averaged over 3 replications per year and 2 years for which the SD was estimated. Straighthead rating of 4 or below was resistant and 6 or above was susceptible.

**Table 4:** Polymorphic SSR markers designed for fine mapping straighthead resistance QTL *qSH-8* on chromosome (chr) 8 using two recombined inbred line (RIL) F_9_ populations.

Marker	Chr	Motif	No.ofRepeats	Physicalpositon(Mb)	Forward Primer	Reverse Primer	Allele size inZhe733,R312 (bp)	Allele size inJing185,Cocodrie (bp)
RM22573	8	atcc	6	5.85	acgagatgggacaagacagatcg	cattgaggcgactgaaagagagg	436,411	436,411
RM22589	8	aaag	5	6.19	tgcatatggtacacacctctttgg	cttgtatgggaggatcggatgg	101,102	101,102
RM22592	8	ag	14	6.20	tgctcctccacctactaccatcc	gcagaaatcttgacagaagagagtgg	185,191	185,201
RM22594	8	atc	16	6.28	cacgacgttgtaaaacgagggtttca	tccatttaaagaaccaaacca	304,297	304,297
RM22613	8	ac	12	6.72	tttctggcccagttcagtacagc	tggtgcgtacatatccctttaacc	236,213	236,213
RM22614	8	ac	13	6.73	ccttgtatcagaagacgcagaagc	actccggatgttcctcaactgc	271,279	271,279
AP3858-1	8	ga	9	6.01	cgttgaggaagccgaggccg	aggaaggtcccaccgaaccct	297,295	297,310

Between RM22573 and RM22589, four out of nine designed InDel markers were found to be polymorphic for Zhe733/R312 (Table 5). In Cocodrie/Jing185 population, *qSH-8* was fine mapped within a 690 kb region between InDel 11 and RM22613 (Fig. 5f). We identified six recombinant RILs in Cocodrie/Jing185 but two had intermediate straighthead ratings 4–6, so they could not be judged as either resistant or susceptibile. Because of the wide *japonica* Cocodrie by *indica* Jing185 cross, sterility due to incompatibility confounded the straighthead ratings in this population.

**Table 5:** Polymorphic InDel markers for fine mapping QTL *qSH-8* on chromosome (chr) 8 using Zhe733/R312 recombined inbred line (RIL) F_9_ population.

Marker name	Chr	Physical distance Mb	InDel size bp	Forward primer	Reverse primer	TM(°C)	Product length bp
InDel 5	8	6.06	7	ggtagaggaaggcgcggcga	agggctagcggcggttgact	55	109
InDel 11	8	6.03	3	tgactgtgctgcaaaggggagt	tcctcaaccattcgcttcgaacac	55	145
InDel 22	8	6.17	3	ctcctcctgctgccacaggtaat	ggcgccacgtaggcagggta	55	240
InDel 27	8	6.14	4	gctcagtggtttctcctgtttgtgg	gctatgagccccaggtttgcca	55	232

In the Zhe733/R312 population, the susceptible RIL12 had the Zhe733 genotype at RM6838 marker but the susceptible R312 genotype in the region from RM22573 to RM72, indicating that a recombination occurred between RM6838 and RM22573, and that *qSH-8* should be downstream of RM6838 (Fig. 5c). Using the same procedure with another susceptible RIL306, the *qSH-8* was restricted to upstream of InDel 27. Analysis of two resistant recombinants, RIL 112 and 174, delimited *qSH-8* to downstream of RM22573. As a result, *qSH-8* was located in a 290 kb interval between RM22573 and InDel 27. Three markers including SSR AP3858-1, InDel 11, and InDel 5 were in the 290 kb interval so should co-segregate with *qSH-8*. Both RIL 12 and 306 had the R312 genotype at AP3858-1, InDel 11 and InDel 5 loci, which matched up with R312 phenotype, susceptible to straighthead with high ratings. Conversely, both RILs 112 and 174 with Zhe733 genotype at these loci had low straighthead ratings.

### Marker Validation

We evaluated the 72 accessions of global germplasm with the InDel 11 marker and identified 30 that had either no alleles or alleles different from the parents, Zhe733, R312, Cocodrie and Jing185. Of the remaining 42 accessions, 3 were heterozygous for resistance, 29 had genotypes which matched the expected phenotype, and 10 accessions were inconsistent (data not shown) (Table 6). For marker AP3858-1, 38 accessions failed to have the parental alleles. Of the remaining 34 accessions, 3 were heterozygous with the resistance, 22 had genotypes which matched the expected phenotype, and 9 accessions were inconsistent (Table 7). Because InDel 5 was monomorphic in Cocodrie/Jing185 population, it was not used to screen the global germplasm collection. *χ^2^* test indicated a high association of InDel 11 with straighthead (*P* = 0.0014), with 76.2% of the genotypes matching the phenotypes among those global accessions (Table 7). Similarly, AP3858-1 was highly associated with straighthead (*P* = 0.0004) with a match of 73.5%. In Zhe733/R312 population, all three markers (InDel 5, InDel 11, and AP3858-1) were verified by *χ^2^* test (*P*<0.0001 for all) where AP3858-1 had a slightly higher ratio of co-segregation (80.0%) than InDel 11 (79.6%) and InDel 5 (78.5%). InDel 5 was not polymorphic in Cocodrie/Jing185 population, but the remaining two markers were verified (*P*<0.0001 for both). InDel 11 had a slightly higher ratio of co-segregation (85.1%) than AP3858-1 (81.2%) in Cocodrie/Jing185 population.

**Table 6:** Association of InDel 11 genotype with straighthead phenotype in a global germplasm collection.

ACP^a^	ACNO^b^	Name	Country of Origin	Allele Size	Genotype^c^	Straighthead rating^d^
PI	629016	Zhe733*	China	151	a	1.2±0.5
PI	615205	Jing185*	China	151	a	2.2±0.5
PI	606331	Cocodrie**	United States	145	b	9.3±0.5
PI	614959	R312**	China	148	b	8.3±0.5
PI	502680	Catibos	Philippines	145	b	8.7±0.5
Clor	12505	PR 433	Puerto Rico	145	b	8.7±0.5
PI	242804	Mojito Colorado	Bolivia	145	b	9.0±0.0
PI	505386	IR 31779-112-1-2-2-3	Philippines	145/151	h	2.7±0.9
PI	596815	376	Cambodia	145/151	h	3.7±2.1
PI	596827	IR-44595	Nepal	151	a	3.0±0.9
PI	281758	Cesariot	France	145/151	h	3.3±0.5
PI	291608	WC 4443	Bolivia	145	b	8.7±0.5
PI	325909	IR 237-20-1	Philippines	148	b	8.7±0.5
PI	331504	IR 547-54-1-2	Philippines	148	b	8.7±0.5
PI	369804	Blakka Tere Thelma	Suriname	145	b	8.7±0.5
PI	392086	CHONTALPA 437	Mexico	148	b	8.7±0.5
PI	392883	Five Months	Guyana	145	b	8.7±0.5
PI	413734	YR 44	Australia	145	b	8.7±0.5
PI	458488	IR 9209-26-2	Philippines	151	a	1.7±0.9
PI	459028	B 541B-PN-58-5-3-1	Indonesia	148	b	8.7±0.5
PI	464599	IR 19759-21-3-3-2	Philippines	151	a	3.0±0.8
PI	584688	CT9901-1-7-M	Colombia	145	b	9.0±0.0
PI	608418	IR 54055-142-2-1-2-3	Philippines	148	b	8.7±0.5
PI	614958	Gui 99	China	151	a	2.3±0.5
PI	615199	Luhongzao	China	151	a	1.3±0.5
PI	615219	Chaoyang No1	China	151	a	2.0±0.8
PI	568890	Adair	United States	145	b	6.5±0.6
PI	643127	Banks	United States	145	b	6.0±0.8
	PVP***	CL 161	United States	145	b	6.8±1.0
PI	634572	KBNT lpa1-1	United States	145	b	7.3±0.5
PI	551950	Mars	United States	145	b	8.0±0.0
PI	636725	Medark	United States	145	b	6.3±1.3
PI	615014	Shufeng 109	China	151	a	1.5±0.6
PI	548630	Wells	United States	145	b	6.0±0.0
PI	614981	XiangzaoxianNo1	China	151	a	1.3±0.5
PI	614966	Zhenshan 97	China	151	a	1.0±0.0

a A total of 42 accessions showed parental alleles screened by InDel 11. The 32 accessions listed above were those that the genotype matched the expected phenotype, whereas for the remaining 10 accessions the genotype did not match the phenotype.

b National Small Grain Collection accessions with PI No. and Clor No.

c ‘a’ as resistant, ‘b’ as susceptible, and ‘h’ as heterozygous genotype but still considered as resistant because straighthead is a dominant trait.

d Straighthead rating using a 1–9 scale with <4 being resistant and >6 being susceptible.

Zhe733 and Jing185*: Straighthead resistant parents for the RIL populations.

Cocodrie and R312**: Straighthead susceptible parents for the RIL populations.

PVP***: Plant variety protection.

**Table 7:** Association of markers with straighthead phenotype using two recombined inbred line (RIL) F_9_ populations and a global germplasm collection including 72 accessions (some germplasm accessions had no parental alleles for a certain marker).

		Resistant lines		Susceptible lines					
Population^a^	Marker name	No of resistant genotype	No of susceptible genotype	No of resistant genotype	No of susceptible genotype	No. of total accessions used for verification^b^	Percent match between phenotype and genotype	χ2	P Value
Global germplasm collection	AP3858-1	7	9	0	18	34	73.50%	18.25	0.0004
	InDel 11	12	8	2	20	42	76.20%	15.53	0.0014
Zhe733/R312 RIL F9 population	AP3858-1	59	9	22	65	155	80.00%	58.02	<0.0001
	InDel 11	60	9	23	65	157	79.60%	49.33	<0.0001
	InDel 5	58	8	24	59	149	78.50%	52.64	<0.0001
Cocodrie/Jing185 RIL F9 population	AP3858-1	24	0	13	32	69	81.20%	32.02	<0.0001
	InDel 11	25	0	11	38	74	85.10%	39.88	<0.0001

a The accessions or RILs selected for marker verification were either resistant with straighthead rating 4 or below or susceptible with rating 6 or above in global germplasm collection and two F_9_ populations.

b A total of 34 accessions were selected for verification of AP3858-1 because remaining 38 had either no alleles of or different from parental Zhe733, R312, Cocodrie and Jing185, and for the same reason, 42 accessions were applied for verification of InDell 11. Hybrid genotypes were included as the resistance group since resistance was dominant over susceptibility.

### Identification of Resistant RILs for Cultivar Development

Although over 40% of the RILs in the Zhe733/R312 cross were rated as resistant to straighthead, this population lacks many of the agronomic and grain quality traits required for cultivar commercialization in the USA. However, the straighthead susceptible parent, Cocodrie, is a widely grown cultivar in the USA [6] and has been used extensively in southern USA breeding programs. Development of improved germplasm having resistance to straighthead in a Cocodrie genetic background will be important for future breeding efforts in the USA. RIL506 from the Cocodrie/Jing185 population had a resistant straighthead rating of 2.3 and shared 101 of the 162 SSR alleles in common with Cocodrie, indicating a genetic similarity of 62% with its Cocodrie parent. Moreover, four other resistant RILs (404, 407, 479 and 480) had a genetic similarity more than 50% with Cocodrie. Thus, these resistant RILs can be used for improving straighthead resistance in long grain *tropical japonica* germplasm pool that is common in the southern USA.

## Discussion

Causal factors for straighthead are not clearly known. Previous studies have associated straighthead with continuous flooding [1], sandy to silt loam textured soils [32], [33], low soil pH and low free iron [34], rich organic matter in soil [35], and minerals of As, Ca, Mn and S [27], [36]. Therefore, there has been no direct way to evaluate for straighthead response either in breeding or research. Instead, the evaluation has been conducted using arsenic-based herbicide MSMA which induces straighthead-like symptoms. However, MSMA has been banned from use in the USA in most crops by the US Environmental Protection Agency because it is a hazardous contaminant to ground water and remains in the soil for a significant length of time [37]. Yan et al. [27] demonstrated that soil application of MSMA results in *As* accumulation in leaves and heading panicles of straighthead susceptible rice plants. Elevated *As* in rice grain as a result of uptake from natural geological formations or from anthropological contaminated sites is of global concern and an area of intensive research [38]–[43]. Therefore, marker-assisted breeding can play an essential role for developing straighthead resistant rice cultivars without sole reliance on MSMA screening.

In this study, we fine mapped a major QTL for straighthead resistance (*qSH-8)* to a 290 kb region and developed three markers to tag *qSH-8* using two F9 RIL populations. *qSH-8* explained 46% of total straighthead variation in one RIL population, and 67% in another. We verified the identified markers for association with straighthead resistance in rice using the two RIL populations and a global germplasm collection. Our results indicate that markers AP3858-1 and Indel11, in particular, can be effectively applied in marker-assisted breeding for straighthead resistance in rice cultivars world-wide.

There are two predominant rice growing regions in the USA, one on the west coast in California with about 18% of USA rice acreage, and another in the south with over 80% of the USA rice production area. The southern USA rice area consists mainly of five states, Arkansas, Louisiana, Missouri, Mississippi and Texas [44]. More than 90% of cultivars grown in the south are long grain type, belonging to the *tropical japonica* sub-population while more than 90% of cultivars in the western area are medium or short grain type, belonging to *temperate japonicas*. From a survey of 1002 germplasm accessions, Agrama and Yan [12] reported only 42 accessions of straighthead resistant germplasm. These resistant accessions originated from 15 countries in 10 geographic regions, with 30 being classified as *indica*, 11 *temperate japonica*, and one that is genetically mixed of *tropical japonica* with *temperate japonica*. This suggests that wide-crosses will need to be used for improving *tropical japonica* long grain rice for straighthead resistance in the southern USA due to a lack of resistant germplasm in this subspecies. Therefore, having markers that are closely linked to this trait will be essential for moving the resistance gene into the *tropical japonica* gene pool without having the phenotype being confounded by sterility due to incompatibility observed in wide crosses.

We evaluated 72 accessions from a global germplasm collection for validation of the fine mapping markers identified in the RIL mapping populations. Among the 42 accessions possessing parental alleles of InDel 11, 12 out of 14 accessions with resistant alleles of Zhe733 (151 bp) and Jing185 (151 bp) had resistant phenotypes with straighthead rating less than 3.7, which resulted in 86% of accuracy for the selection. For AP3858-1, 7 accessions with the resistant alleles of Zhe733 and Jing185 were all phenotypically resistant with straighthead rating less than 3.7, which resulted in 100% of accuracy for the selection. Thus, elimination of the progenies which have no resistant alleles of both AP3858-1 and InDel 11 should be effective, and both markers could be referenced each other in a breeding program. In addition, our study identified resistant RILs that had over 50% of genetic similarity with Cocodrie, a cultivar that has gained wide acceptance in the southern USA. These RILs will be valuable to improve straighthead resistance of *tropical japonica* cultivars without having to rely upon unadapted germplasm as a resistant resource.
